# *Trachelomonas bituricensis* var. *lotharingia* M.L. Poucques 1952, a morphologically interesting, rare euglenoid new to the algal flora of the Czech Republic

**DOI:** 10.3897/phytokeys.61.7408

**Published:** 2016-02-25

**Authors:** Josef Juráň

**Affiliations:** 1 Department of Botany, Faculty of Science, University of South Bohemia, Na Zlaté Stoce 1, České Budějovice CZ-37005, Czech Republic; 2 Centre for Phycology, Institute of Botany AS CR, Dukelská 135, Třeboň CZ-37982, Czech Republic

**Keywords:** Czech Republic, floristics, microalgae, *Trachelomonas*

## Abstract

This report describes the discovery of the rare euglenoid taxon Trachelomonas bituricensis var. lotharingia in a small mesotrophic pond in the Czech Republic. Only limited data are available on the distribution of this taxon as same as for typical variety of *Trachelomonas bituricensis*, even though this taxon is morphologically very well defined. I provide a brief discussion of the taxonomic validity of this taxon based only on morphological features, which are characteristic for the taxonomy of the genus *Trachelomonas*. This finding is completely new for the algal flora of the Czech Republic. This report provides new information about the worldwide distribution of this taxon and its ecology.

## Introduction

Floristics studies have had a long tradition in the Czech Republic beginning in the second half of the 19th century, when several works about alga flora in this country were published. Since the publication of pioneering works by Kirchner (1878) and Hansgirg (1892, 1899), Lhotský and Rosa (1955) published a list of algal and cyanobacterial species from the Moravia region. The current knowledge about algal diversity in this region was summarised by Poulíčková et al. (2004). All of these reports describe floristic studies examining the prevalence of all groups of algae in our state. These studies, combined with recent reports about the distribution of various groups of algae (e.g., Caisová and Gąbka 2009, Kaštovský et al. 2010, Kučera et al. 2008 and Šťastný 2010), provide comprehensive information about algal flora. Unfortunately, little is known about several of these groups and their distribution, e.g., diatoms, dinophytes and euglenoids.

I performed a nearly 5 year survey during my bachelors and masters studies investigating the diversity of photosynthetic euglenophytes (Juráň 2010, 2012) based on extensive literature reviews and my own floristic studies across the country. As a result, I constructed a preliminary checklist of euglenoid taxa of the Czech Republic including several species that were newly reported in our state. This checklist will be published soon. One of these new species is Trachelomonas bituricensis var. lotharingia from Ďáblík pond in South Bohemia.

The genus *Trachelomonas* includes euglenoids with their cells enclosed in envelopes (loricas) comprising polysaccharides with a content of iron and several inorganic compounds (Pereira et al. 2003). These loricas are highly variable in shape and surface morphology. Listed features are traditional markers used in *Trachelomonas* taxonomy, as proposed by Deflandre (1926). Since the original description of *Trachelomonas* by Ehrenberg (1834), more than 1700 taxa have been identified due to this high morphological variability in the genus (Guiry and Guiry 2015).

*Trachelomonas bituricensis* was originally described by Wurtz (1947), who identified this species in plankton from the pond of the Hardouine in La Brenne, France. In the typical species, the lorica is covered with long, sharp, curved spines. One variety of this species, Trachelomonas bituricensis var. lotharingia, was first described by Poucques (1952). The description of new taxon was based on material from a wetland in the Woëvre region in France. This variety has shorter collars (see Fig. 3) than the typical forma described by Wurtz (1947). In addition, the edge of the collar is more undulated.

## Methods

**Figure 1.**
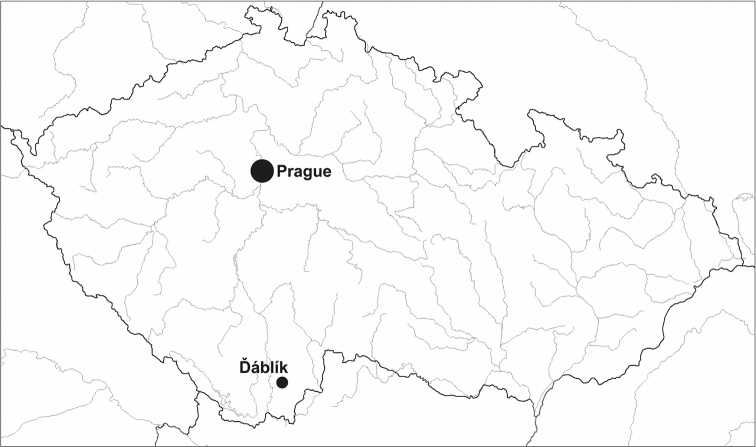
Location of the studied locality.

**Figure 2.**
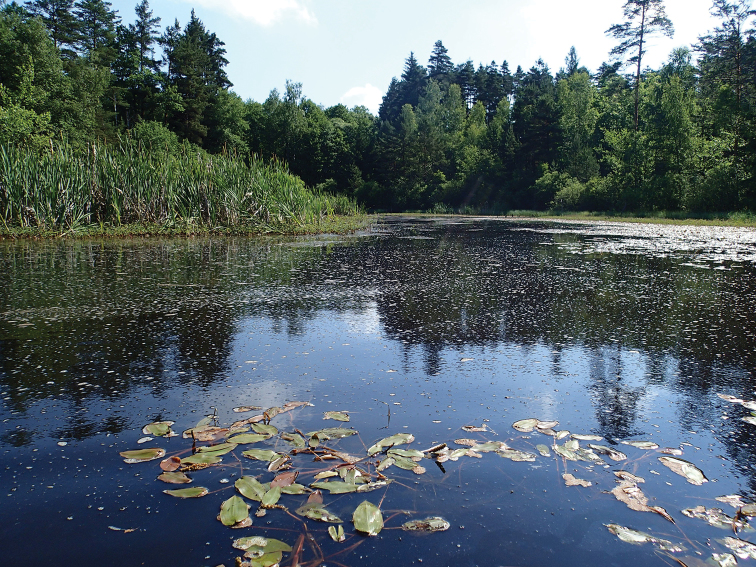
Photograph of a free water surface with knotweed (*Potamogeton* sp., at the front) and *Carex* spp., together with *Sphagnum* spp. communities (left).

The aim of this study was to survey the algal flora of the small mesotrophic pond Ďáblík (South Bohemia region, Czech Republic, 48°49'41.0"N, 14°35'49.6"E, see Figs 1 and 2). This habitat is a small pond that functions as a primary retention pond, without fishery management. This pond contains huge communities of *Carex* spp. and *Sphagnum* spp. and a stable population of the charophycean alga *Nitella flexilis*. The pond is progressively becoming overgrown with vegetation. The locality is favourable for the growth of threatened vascular plants, e.g., *Nymphaea candida*, *Calla palustris* and *Menyanthes trifoliata*. Wetland plants *Utricularia australis* and *Potamogeton obtusifolius* are common in this locality (Albrecht 2003).

**Figure 3.**
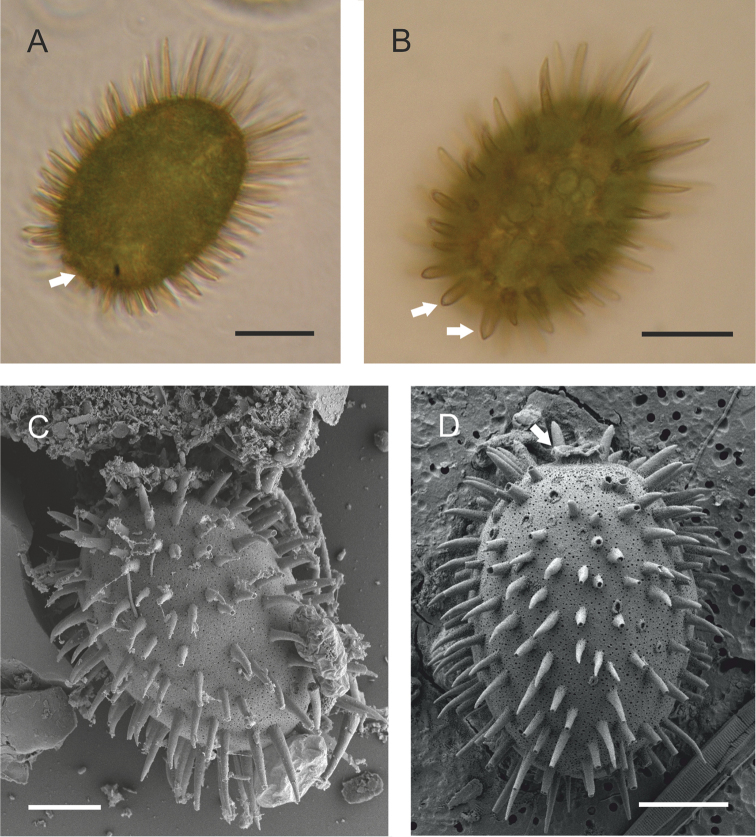
Visualisation of the morphology of Trachelomonas bituricensis var. lotharingia by optical and electron scanning microscopy. **A** Overall appearance of lorica under an optical microscope, with a short collar with undulated edge (marked with arrow) **B** Detailed image of the lorica surface with three types of spines: long, curved spines at the antapical part of the lorica; shorter spines on the lorica’s body; and several straight spines near the apical pore (marked with arrows). Note the numerous disc-like chloroplasts in the cell **C, D** Loricas viewed by scanning electron microscopy, with clearly visible punctuation at the surface, and short collar with undulated edge (marked with arrow). Scale bars = 10 μm.

In 2012 and 2013, a pilot survey was taken, and in 2014, a more detailed survey with monthly sampling at five chosen locations was performed. The aim of this survey was to record algal diversity, especially for loricate euglenoid genera *Trachelomonas* and *Strombomonas*. Samples were taken using a 20 µm plankton net, and the material was fixed in formaldehyde solution and Lugol’s iodine solution. Samples were examined microscopically under an Olympus BX51 microscope and photographed with an Olympus DP-71 camera with DP Controller 3.1.267 software. Samples fixed in iodine solution were used for scanning electron microscopy. Materials for scanning electron microscopy were prepared as described by Conforti (2009). The material was filtered through polycarbonate membrane filters (1 μm pore size) and air-dried. Filter pieces were attached to stubs and subsequently coated with gold. The specimens were then saved for future scanning electron microscopy analysis or examined under a JEOL JSM-7401F scanning electron microscope (Institute of Parasitology, Biology centre of AS CR, České Budějovice).

## Results

The algal flora of the Ďáblík pond is dominated by dinophytes (*Peridinium* spp.), together with desmids and metaphytic communities of *Spirogyra* spp. steril., as well as diatoms (especially *Gomphonema* species). Taxon-rich flora of the loricate euglenoids will be studied in detail using optical and scanning electron microscopy in conjunction with comparisons of the chemical and physical parameters of pond water. Based on the preliminary results, some of the taxa appear to represent new algal flora of the Czech Republic, but these taxa require further study. The most interesting finding is the discovery of Trachelomonas bituricensis var. lotharingia, a newly recorded variety of algal flora in the Czech Republic. This variety is quite common in Ďáblík pond, which contains a stable population of this alga. This finding represents the first record of this taxon among algal flora of the Czech Republic based on a detailed literature review and the floristic survey across the Czech Republic that I performed during my bachelors and masters studies (Juráň 2010, 2012).

The loricas in this species are (41 –)45–46 µm in length and (30 –)33–35 µm wide. The cell contains numerous disc-like chloroplasts, probably without pyrenoids. The surface of the lorica in this taxon from Ďáblík pond is punctate and covered with well-developed hollow, sharp spines (see Fig. 3A–D), most of which are slightly curved and cover the body of the lorica. The caudal region contains very long, curved spines, and only a few narrow, conical spines surround the apical pore, which has a collar (see Fig. 3B, marked with arrows). The collar of the lorica is short, with an undulated edge (see Fig. 3D, marked with arrow). This taxon has always been found during the spring at shallow sites with populations of *Nitella flexilis* near the banks of the pond. These findings suggest that Trachelomonas bituricensis var. lotharingia is probably a benthic or metaphytic taxon.

A comparison of a typical variety of *Trachelomonas bituricensis*, the variety described by Poucques (1952) and the material found in Ďáblík pond is presented in Table 1.

**Table 1.** Comparison of morphological data about *Trachelomonas bituricensis* taxa.

Feature	Wurtz (1947)	Poucques (1952)	This study
**Shape**		ellipsoidal to ovoid	ellipsoidal
Length*	40 µm	40 µm	(41 –)45–46 µm
Width*	20 µm	30 µm	(30 –)33–35
**Spine length**			
- **conical spines**	N/A	4–6 µm	4–5 µm
- **posterior part**	N/A	12–14 µm	8–13 µm
**Collar**	slightly enlarged, with thickening at the base	short, large, regular undulated edge	short collar with undulated edge
**Chloroplasts**	N/A	N/A	numerous, discoid
**Ecology**	fishpond; plankton	wetland; plankton	mesotrophic pond; benthos, metaphyton

* without spines

## Discussion

These two taxa (*Trachelomonas bituricensis* and Trachelomonas bituricensis var. lotharingia) are commonly mentioned in works about euglenophytes (e.g., Huber-Pestalozzi 1955, Popova 1966, Starmach 1983 and Vetrova 1986). These taxa have distinct morphology that is easy to distinguish from that of other species, the descriptions of which are commonly used in taxonomic keys. However, data about the occurrence of these taxa are very limited. A typical form of *Trachelomonas bituricensis* was only recently reported to occur in several places, including benthos in a fishpond in Poleski National Park, Poland (Kočárková et al. 2002) and two rivers in Kosovo (Kurteshi et al. 2013a, 2013b). The second reported location of *lotharingia* variety was a bog in the Russian taiga, where this taxon was found in a wetland containing saw grass (*Carex* sp.) (Safonova 1987). In addition, Vetrova (1986) identified this variety among algal flora of Ukraine. Perhaps the most recently reported occurrence of this taxon was in the Southeastern US, where it was rarely observed in ditches, lakes and ponds (Wolowski and Walne 2007).

Several species of *Trachelomonas* have the similar morphology as *Trachelomonas bituricensis*. The most resembling taxa are *Trachelomonas horrida*, which was first described by Palmer (1905), as well as *Trachelomonas megalacantha* (DaCunha, 1914) and *Trachelomonas spectabilis* (Deflandre, 1926). All of these species have loricas covered with well-developed long, sharp spines, but their spines are straight, unlike the curved spikes on the loricas of *Trachelomonas bituricensis*. Huber-Pestalozzi (1955) argued that Trachelomonas bituricensis var. lotharingia is highly similar to *Trachelomonas spinosa*, which is found in ponds containing aquatic plants (Stokes 1890), and that it probably represents a larger form of these taxa. Stokes’ (1890) description of the surface and shape of the *Trachelomonas spinosa* lorica is similar to that of *Trachelomonas bituricensis*, whereas its collar is described as a “… *short, smooth, truncate extension*”, with no mention of the undulated edge or additional cylindrical spines. In addition, Tell and Conforti (1986) described *Trachelomonas spinosa* from Argentina as having a similar morphology, with an apical pore with a short, thick neck surrounded by spines; again, an undulated edge is not mentioned. Vetrova (1986) noted the similarity between *Trachelomonas bituricensis* and *lotharingia*-variety with *Trachelomonas horrida* and *Trachelomonas spinosa*, although they did not mention the taxonomy of *Trachelomonas bituricensis* taxa in a subsequent report and the validity of this variety could be discussed. Finally, Wolowski and Walne (2007) reported that Trachelomonas bituricensis var. lotharingia from the US has a dentate collar.

The high morphological variability of the genus *Trachelomonas* makes classification based only on morphology (e.g., shape, surface, absence or presence of various forms of collar and sometimes colour of the lorica) quite difficult and has led to the description of morphologically similar forms as separate taxa. As a result of morphological variability, the traditional taxonomy of *Trachelomonas* based only on a description of lorica characters is problematic for several reasons. First, there are several examples of the morphology of euglenoids changing depending on environmental conditions. This variability in shape was observed in several euglenoid genera in culture (Conforti 1998) and those cultivated in situ under natural conditions (Bauer et al. 2012). The cells of these taxa exhibited morphological changes depending on the level of organic carbon in the medium or the environment. Pringsheim (1953) and Singh (1956a, b) found that in some *Trachelomonas* species, the loricas of cells in clonal culture (i.e., descendants of a single cell) have morphological diversity in shape and surface, which probably depends on their specific ontogeny. This diversity represents the main problem with classification based solely on morphology. Indeed, in discussing *Euglena* taxonomy, Pringsheim (1956) noted that, “Certain authors could not resist the temptation to give a name to every minor deviation from the ‘type’ previously described or prevailing at the same or at similar places”. This comment accurately describes the state of taxonomy of the genus *Trachelomonas*. Therefore, the large number of currently described taxa within the genus *Trachelomonas* likely reflects the morphological variability created by environmental conditions. Many of these taxa may instead be recognised as “eco-morphs” of the typical form. Furthermore, Pringsheim (1953, 1956) claimed that unless individual species are cultured separately, the effects of external factors affecting heritable morphological traits cannot be distinguished. In light of these findings, perhaps Trachelomonas bituricensis var. lotharingia is only an eco-morph of the typical forma, or perhaps it represents a *bituricensis* eco-morph of *Trachelomonas spinosa*. Alternatively, this alga may represent a complex of species. More detailed morphological studies are needed to solve this taxonomic puzzle, including basic morphometric analysis and especially molecular analysis.

Many reports about euglenoid taxonomy based on morphological and molecular data (e.g., Karnkowska-Ishikawa et al. 2010, 2011, 2012, 2013 and Kosmala et al. 2005, 2007a, 2007b, 2009) show that several species or other intraspecific taxa may instead only represent eco-morphs or ontogenetic stages of individual species, as discussed in a report about *Monomorphina* genus taxonomy (Kosmala et al. 2007b). Unfortunately, similar studies about the taxonomy of the genus *Trachelomonas* remain to be performed. Several recent works (e.g., Nejmová et al. 2011, Škaloud et al. 2015 and Veselá et al. 2012) about species concept in algae reflect a combination of molecular and morphological data. Therefore, a combination of ecological, morphological and molecular data can be used to define the species concept of a genus with worldwide distribution such as *Trachelomonas*. However, this issue requires further study.

## Conclusion

This report describes what is likely the third record of Trachelomonas bituricensis var. lotharingia in Europe and the first record of this taxon in the Czech Republic. The description of this taxon is morphologically and ecologically similar to the original description. The taxonomic validity of this taxon remains to be validated, as it may represent part of a complex of morphologically similar species together with *Trachelomonas spinosa* and *Trachelomonas horrida*. Nonetheless, the discovery of this taxon sheds light on the distribution of algae of the Czech Republic and European algal flora in general.
